# Inductive and Deductive Approaches to Acute Cell Injury

**DOI:** 10.1155/2014/859341

**Published:** 2014-10-13

**Authors:** Donald J. DeGracia, Fika Tri Anggraini, Doaa Taha Metwally Taha, Zhi-Feng Huang

**Affiliations:** ^1^Department of Physiology, Wayne State University, 4116 Scott Hall, 540 East Canfield Avenue, Detroit, MI 48201, USA; ^2^Department of Physics and Astronomy, Wayne State University, Detroit, MI 48201, USA

## Abstract

Many clinically relevant forms of acute injury, such as stroke, traumatic brain injury, and myocardial infarction, have resisted treatments to prevent cell death following injury. The clinical failures can be linked to the currently used inductive models based on biological specifics of the injury system. Here we contrast the application of inductive and deductive models of acute cell injury. Using brain ischemia as a case study, we discuss limitations in inductive inferences, including the inability to unambiguously assign cell death causality and the lack of a systematic quantitative framework. These limitations follow from an overemphasis on qualitative molecular pathways specific to the injured system. Our recently developed nonlinear dynamical theory of cell injury provides a generic, systematic approach to cell injury in which attractor states and system parameters are used to quantitatively characterize acute injury systems. The theoretical, empirical, and therapeutic implications of shifting to a deductive framework are discussed. We illustrate how a deductive mathematical framework offers tangible advantages over qualitative inductive models for the development of therapeutics of acutely injured biological systems.

## 1. Introduction

In spite of decades of intensive research and hundreds of clinical trials, clinically important, life-threatening forms of acute injury have stubbornly resisted successful therapeutic intervention. Two notable examples are ischemia of the brain [1] and ischemia of the heart [2]. The former manifests clinically during stroke and cardiac arrest and the latter during myocardial infarction. The clinical trial failures have prompted three main responses: (1) some have closely scrutinized technical details of preclinical and clinical research [3, 4]; (2) others have advocated for additional basic research [5]; (3) still others have suggested therapeutics may not be possible for a given type of injury, such as stroke [6]. The first option focuses on analytical techniques, the second emphasizes empirical information, and the third blurs the distinction between the science of acute injury and its technological application in the form of therapy. The area given the least consideration in the literature evaluating clinical trial failures is the theoretical foundation [1].

We recently developed a nonlinear dynamical theory of acute cell injury [7] that suggests that an important aspect of clinical trials failure can be attributed to inadequate theory. Our purpose here is to discuss that the theoretical problems can fruitfully be encapsulated by the classical distinction between inductive and deductive reasoning. The nonlinear cell injury model is a deductive framework for understanding cellular injury in a generic fashion. The approaches used in mainstream biomedicine are inductive and based on the biological specifics of the injury system under study. However, inductive phenomenological approaches do not allow a strict attribution of causality. Without a clear concept of cell death causality, how is therapy, the prevention of cell death, supposed to be carried out? Further, inductively studying phenomenology fosters a qualitative mind-set that neglects systematic quantitative considerations. Clinical injuries occur across a range of injury magnitudes and cell responses are generally graded [8]. Thus, how are cellular responses, causal mechanisms, and outcome to be linked in a quantitative fashion to injury magnitude? Inductive and deductive models possess different implications for conceptualizing causal mechanisms and quantification of injury states. We build the case that a deductive, mathematical framework offers tangible advantages over qualitative inductive models for the development of therapeutics of acutely injured biological systems.

## 2. Induction and Deduction

Here we briefly discuss the strengths and weaknesses of induction and deduction. Induction reasons from the specific to the general. Many specific cases are observed, and from these general conclusions are drawn. Such conclusions are called “inductive inferences”. Deduction is the opposite of induction. With deduction one begins with general premises and from these deduces, or derives, specific cases. Both forms of reasoning are used in modern science, but their differences have long bothered commentators of science.

There is asymmetry between induction and deduction with respect to preserving the truth value of propositions. Truth value can be binary, that is, true or false, or it can be graded when probabilities are associated with the occurrence of events, that is, something is 33% probable under given conditions. Deductive reasoning explicitly exposes the logical steps and thereby preserves the truth value from premises to conclusions. The canonical example of deduction is the derivation of a theorem from a set of axioms in mathematics. Induction, on the other hand, generalizes based on past events “the sun always rises in the East, and therefore will rise in the East tomorrow” or generalizes from specific observations “all observed pieces of copper conducted electricity, and therefore all copper conducts electricity”. Unlike deduction, there cannot be a finite, explicit series of logical steps associated with an inductive inference because these are always based on open-ended sets, such as extrapolating from past to future events, or from observed cases to unobserved cases.

In general, deduction is used in mathematics and physics where premises can be expressed mathematically. Then, derivation of theorems or solutions of equations reveal consequences that follow inevitably from the premises. A weakness of the scientific application of deduction is that the axioms must accurately represent the system under study. If the axioms are incorrect so will be the conclusions, even if the intervening steps are logically sound. This weakness is offset by the flexibility with which one can alter the axioms and rederive new conclusions accordingly. An example of successful deduction is Einstein's special theory of relativity that is based on only two axioms [9]: (1) the laws of physics are the same for all observers in inertial reference frames, and (2) the speed of light in a vacuum is constant. Both axioms are expressible mathematically, and the resulting deductions, such as length contraction at high velocities, have been successfully verified by experiment [10].

Sciences seeking to explain complex phenomena, such as biology or sociology, have traditionally utilized induction more than deduction. One example of the application of inductive logic in biology is Darwin's theory of evolution by natural selection [11]. This theory, or model, was not derived from axiomatic principles but instead was a generalization drawn from many specific pieces of evidence: comparative anatomy, comparative ethology, Darwin's field observations in the Galapagos Islands, domestic breeding experience, and other evidence went into the inductive inference that species arise by natural selection. Given the generic weaknesses of induction, it is interesting to note that deductive alternatives to explaining biological form are emerging in biology [12].

The analysis of induction is associated with the work of the philosopher David Hume. Hume demonstrated that deduction cannot be used to explicitly prove the truth value of an inductive inference. The inductive inference to be proved must be taken as an axiom, thereby leading to circular logic between the premise and conclusion [13]. Thus, inductive inferences are open-ended, and acting upon them requires faith that no contradictory case will eventually appear. An important implication of inductive inference is that it does not, strictly speaking, allow attribution of causality. This conclusion has bothered philosophers of science who have sought to wiggle out of Hume's problem of induction.

A summary of philosophy of science views on induction [14] is certainly beyond our present scope, so here we mention only two salient ideas on this intellectual landscape. Karl Popper suggested that science can avoid the weaknesses of induction by combining observation and deduction in the quest to falsify hypotheses [15]. In this view, confirmation or verification (i.e., seeking evidence to prove a hypothesis is true) is discouraged, and experiments should be designed to expose if an idea is false. Popper's thinking underlies, for example, the practice of disproving the null hypothesis in statistics. However, Popper's view was superseded by Kuhn's model of scientific paradigms as complex webs of belief that cannot easily be shoehorned into the classical distinction of inductive versus deductive reasoning [16]. The net result is that the use of induction in science has not been resolved and remains an open question.

The complexity of the world has necessitated the use of induction for systems that cannot be deduced from “first principles.” Therefore induction and deduction have coexisted in their respective domains and represent a significant gulf between approaches traditionally applied in, for example, biology and physics, respectively. However, continuing advances in mathematics, physics, and the understanding of complex systems have expanded the purview of deductive logic into traditionally inductive realms, including biology and the corollary study of biology, biomedicine. Before discussing a deductive approach to biomedicine, we illustrate the prevailing inductive biomedical approach using the field of brain ischemia as an example.

## 3. Brain Ischemia

While we use brain ischemia as our case study of inductive biomedicine, the template we derive below holds for other biomedical fields such as heart ischemia, traumatic brain injury, epilepsy, diabetes, acute kidney injury, cancer, and other complex disease states. A decrease or cessation in blood flow to any organ is called “ischemia.” Brain ischemia can cause neurons to die, even in cases where blood flow resumes (called reperfusion). The scientific problem is how does ischemia cause neurons to die? The biomedical problem is can the neuron death be prevented?

There is massive clinical experience with brain ischemia in the forms of stroke and cardiac arrest and resuscitation. Stroke is cessation of blood flow to a specific part of the brain (called “focal brain ischemia”) due to a focal occlusion in approximately the brain vascular tree. Stroke affects approximately three-quarter million people in the USA each year and is the fourth leading cause of death [17]. Cardiac arrest stops all blood flow to the brain and is an example of global brain ischemia. Out-of-hospital cardiac arrest occurs in >350,000 people a year in the USA [18] and only 11% of them survive. Between 66% and 75% of cardiac arrest victims die from brain death due to ischemia [19, 20]. Thus, brain ischemia results in substantial mortality and morbidity.

It was estimated that stroke costs $43 billion in direct and indirect costs in 2010 in the USA [21]. It is no surprise that this clinical challenge has been met with a proportional research response. At NIH, the National Institute of Neurological Disorders and Stroke (NINDS) studies brain ischemia and other brain disorders. The 2014 budget of NINDS was $1.6 billion. In 2010, American Heart Association spent $110 million on cardiovascular research [22], including stroke, which increased to $129 million in 2012 [23]. These tremendous research efforts have had positive impact at the front end of the disease process: incidence of heart disease and stroke has progressively decreased over the past decade [24]. However, at the back end, after a patient has suffered brain ischemia, all the research to present has produced minimal clinically tangible benefit. Physicians have only limited options to treat ischemia-induced neuron death in stroke and cardiac arrest patients (e.g., tPA [25] and therapeutic hypothermia [26], resp.), and these options can only be applied to a small subset of brain ischemia patients [27]. Therefore, with respect to preventing neurological damage after the injury had occurred, it is fair to conclude that the amount of research invested over the past decades has not produced a proportional return on investment. We suggest that a key factor is that brain ischemia research has been theoretically rudderless. To build this case, we first summarize the salient points in the evolution of the field to illustrate its inductive logic.

## 4. Brain Ischemia Studies: From Parameters to Rote

The earliest approaches to brain ischemia studies were empirically systematic but were not grounded in any deductive framework. Early investigations identified an important concept of “thresholds,” referring to the fact that specific phenomena occur in the brain as a function of both the degree of reduction of blood flow and its duration [28–30]. For example, a 50% reduction in brain blood flow for about 30 min triggers stress responses in neurons, such as the heat shock response but has minimal effect on the electrical conductive properties of the neurons [31]. However, at a threshold of ~20% brain blood flow, neuronal ATP levels decrease and neurons become electrically silent [31]. Blood flow below 20% for any substantial length of time (e.g., ~≥30 min) causes neurons to die [31]. Thus, neuron death was discovered to be dependent on (1) the degree and (2) the duration of blood flow reduction. Both factors are, by current standards, system parameters, but the field did not evolve in the quantitative direction implied by this insight.

Because there are so many possible combinations of blood flow reduction and time, it is impossible to empirically evaluate all of them. Thus, the field has standardized on specific blood flow reduction and time increments. This standardization on specific parameter values was predicated on the development of animal models of brain ischemia which mimic features of human brain ischemia [32, 33]. The study of focal ischemia, which models stroke injury, focused on examining 100% flow reduction in the middle cerebral artery unilaterally, with or without resumption of blood flow. When blood flow is not resumed it is called “permanent focal ischemia” and when blood flow is resumed (usually after 1-2 hr ischemia) it is called “transient focal ischemia.” Thus, the qualitative terms replaced the quantitative parameters underlying these conditions. Via the animal models, stroke researchers identified two forms of ischemia-induced neuron death: necrosis of tissue in the ischemic epicenter and a delayed form of neuron death in a penumbral volume around the epicenter [34–36].

Studies of global brain ischemia, modeling cardiac arrest, followed a similar progression. Global brain blood flow can range from normal brain blood flow (100%) to zero blood flow. The duration of global brain ischemia must be compatible with the organism remaining alive (~30 min for zero global brain blood flow). In the 1980s, Kirino discovered that specific durations of global brain ischemia caused a delayed neuronal death of a specific subtype of hippocampal neurons (CA1 layer) days after the ischemia [34, 37]. Again, the field standardized on ischemia conditions causing CA1 death and focused on the question: what causes the CA1 neurons to die in a delayed fashion days after global brain ischemia?

In summary, the original studies of brain ischemia began in a reasonably systematic fashion studying the effects of ranges of brain blood flow and duration. But as the field matured, the experimental animal model systems became standardized around only a few parameter combinations of brain blood flow and duration. These were never conceptualized as system parameters in a formal deductive sense but instead evolved into the rote procedure for conducting the experimental animal models. The animal models have been used to dissect brain tissue after ischemia to investigate how it changes compared to the nonischemic brain. The observed phenomenological differences became conflated with causation.

## 5. Neuroprotection: The Holy Grail of Brain Ischemia Studies

Before detailing phenomenological studies, we summarize the therapeutic expectation for understanding how ischemia causes neurons to die. The brain uses a disproportionate amount of metabolic energy: 20% of cardiac output feeds the brain, which is only 2% of body mass. The brain is completely dependent on blood flow for making ATP and has little or no backup reserves of glucose [38]. The rate of ATP loss depends on the type of ischemia (focal or global) and the animal model used, but generally ATP levels are less than 15% of control after 2 min in global ischemia and 25%, or substantially less, after 10 min of focal ischemia [34].

Thus, the proximal damage event in brain ischemia is rapid loss of ATP. Clinically, this gives rise to two treatment possibilities: (1) reperfusion therapies and (2) neuroprotection. In cultured brain slices subjected to oxygen and glucose deprivation, ATP levels renormalized within ~10 min after resumption of glucose and oxygen [39]. Therefore, reperfusions therapies seek to rapidly regain blood flow and thereby reduce the ischemia as quickly as possible to minimize loss of ATP [40]. Reperfusion therapies can be administered surgically or chemically (with tPA) and are used clinically [41]. However, reperfusion therapies can only be applied under limited circumstances. In the majority of cases, the patient experiences brain ischemia in a setting where there is no possibility of performing a reperfusion therapy. In such cases the ischemia is unavoidable and occurs for some duration before the patient receives medical attention. In the case of stroke, this may be 24 hours after the ischemic event has occurred [42]. For cardiac arrest, emergency medical service response time is often the limiting duration, which is on the order of 5–10 minutes in most major cities [43].

Given that some neurons will not die for days after the ischemic event, it is possible to imagine a therapy that will prevent delayed neuronal death. This hypothetical therapy is called “neuroprotection” [44] and is the holy grail of brain ischemia research. One would like to know* what causes* the neurons to die in a delayed fashion. Then, one could, in principle, inhibit this cause and prevent the death of the neurons.

## 6. An Inductive Jungle of Causes

Here we outline some of the biological observations specific to neurons which have experienced ischemia. There have been, for roughly the past 30 years, continuous attempts to conflate this phenomenology with cell death causation. This has led to a repetitive behavior where a specific biological event is treated as causative, and drugs that inhibit this event are investigated in the preclinical arena, some of which proceeded to human clinical trials. All these attempts have failed to show improvement in outcome.

The loss of ATP has many effects in neurons that are widely believed, incorrectly [45], to form a “cascade,” a linear sequence of events [46–48]. All explanations of how ischemia causes neuronal death begin with loss of ATP, but then the explanations exponentially multiply and today form an indecipherable quagmire of qualitative biological pathways. Here we only briefly review early observations that today are the core of the “ischemic cascade” concept.

An influential paper in the early 1980s posited a key role for Ca^2+^ influx in ischemic damage to neurons [49]. Empirical measurements showed drops in extracellular [Ca^2+^] [50] and increases in intracellular Ca^2+^ [51]. Distinct intracellular pathologies were inferred to be trigger from this “Ca^2+^ overload” that could plausibly cause neurons to die [52]. However, clinical trials with Ca^2+^ channel blockers failed to improve outcome [53]. For a variety of empirical reasons, the “Ca^2+^ overload” theory morphed into the “excitotoxicity” theory [54, 55], where overactivation of glutamatergic NMDA receptors were thought responsible for Ca^2+^ overload. Clinical trials with NMDA receptor antagonists also failed to improve outcome [1] and, given the central role of glutamate in brain neurotransmission, were accompanied by undesirable side effects [56].

By the early 1990s, free radical biology became widely recognized as a means of killing cells. Many studies in animal experimental models showed definitively that a number of different free radical species form in postischemic neurons [57]. However, clinical trials of free radical scavengers have failed to improve outcome in human stroke patients [1, 58, 59].

In addition to excitotoxicity and oxidative stress, other neuronal pathways have garnered attention as possible causes of cell death: nitric oxide, intrinsic and extrinsic apoptosis, ubiquitin-proteosome, sumoylation, autophagy, and so on (many of these are reviewed in [34]). There is evidence of activation of each pathway and evidence that inhibition of each pathway can decrease cell death in experimental animals. Investigations of causes of cell death have extended beyond neuron molecular biology to encompass the “neurovascular unit”, including vascular endothelial responses, astrocytes, oligodendrocytes, and various immune system responses [60]. There is evidence that all of these systems play a role in postischemic brain responses.

Blocking any number of these factors can prevent neuron death in experimental animal models. However, many of these strategies have been tested in human clinical trials, and literally all have failed. Some of the specific agents tested in clinical trials were (as taken from [1]): NMDA and AMPA glutamate channel blockers, Na^+^ and K^+^ channel blockers, benzodiazepine, opioid antagonists, anti-inflammatory agents, immunosuppressants, free radical scavengers, growth factors, antiapoptotic agents, an angiotensin-1 receptor antagonist, an alpha2 adrenoceptor agonist, a beta-adrenergic blocker, an endothelin antagonist, an antiplatelet agent, anticoagulants, antithrombotics, a phosphodiesterase inhibitor, a serotonin antagonist, hemodilution agents, an NO donor, and others.

The above list of agents tested in clinical trials was chosen based on what is widely considered the “theory” of brain ischemia research, a scheme known as the “ischemic cascade” [47, 48, 61–63]. The “ischemic cascade” is the attempt to depict the many cell and molecular level events that occur in postischemic neurons. We do not attempt to represent this here due to its sheer qualitative complexity. The above discussion and the list of clinical trial agents above are meant to convey a sense of the variety of molecular elements posited in this scheme.

Because these events occur downstream from the loss of ATP during ischemia, or downstream from reintroduction of oxygen with reperfusion, the qualitative changes have been construed as a cascade, a term which implies a linear sequence of causal events. The term “cascade” derives from a loose sense of the time progression of various elements discussed above. However, many, of these intracellular reactions and cell responses proceed in parallel, which is the antithesis of a cascade. Thus, the “ischemic cascade,” as an intellectual construct, is not truly an accurate portrayal of the real complexity found in postischemic brain tissue. The attempts to symbolically depict these changes more resembles a patchwork quilt upon which new pathways are constantly being grafted. Since the basic unit of the “ischemic cascade” scheme is the molecular pathway, we next discuss molecular pathway diagrams.

## 7. The Problems with Pathways

A molecular pathway is intended to provide a causal explanation of how ischemia kills neurons. The logic then is that inhibition of the pathway should prevent the cell from dying. Pathways are typically depicted in the form A → B → C. A is a proximal ischemia-induced event such as the loss of ATP, or increased intracellular Ca^2+^. C is some marker measured in the postischemic tissue, whose presence correlates with the eventual disappearance of neurons. B is a linker that provides an ostensibly causal connection between A and C, usually consisting of multiple substeps. Thus the “ischemic cascade” can be seen as the accumulated result of the field investigating various internal parts of the cell, one by one, where the parts of the cell are conceptualized by an A → B → C pathway, presumed to cause the neuron to die. This is an inductive approach because no axiomatic principle guides this search. Instead, it is based on the specific details of neuron molecular biology as these emerge from the basic neurosciences.

**Figure 1:**
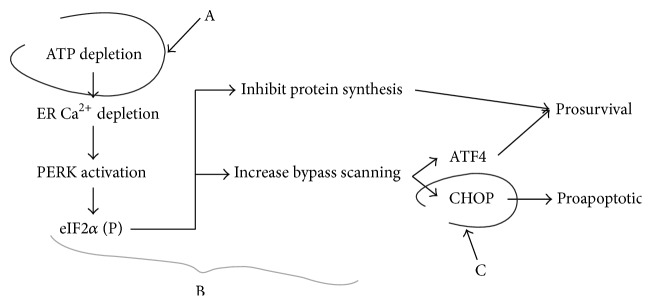
Example of application of inductive logic to attribute causality to cell death after brain ischemia. In the “A → B → C” notation, A is the depletion of ATP accompanying ischemia. The cell death marker C is the protein CHOP (C/EBP homology protein, where C/EBP stands for caat-enhancer-binding protein. CHOP is also called GADD153). The B linker is a set of binding interactions that constitute part of the unfolded protein response, an ER stress response.

We illustrate the limitations of the pathway approach by an example, with a pathway used by one of the authors in past work (Figure 1) [64, 65]. Here, the endoplasmic reticulum (ER) stress response known as the unfolded protein response (UPR) is partially illustrated and used to inductively model how the UPR leads to either cell survival or death [66]. In this instance step A is loss of ATP, marker C is the presence of the prodeath protein CHOP (GADD 153), and the intervening steps (B) provide links between loss of ATP and the presence of CHOP in the postischemic neurons.

While appearing plausible on first glance, a more critical look reveals serious flaws with this way of thinking. First, the symbolism of pathway “A → B → C” represents nothing more than interacting molecular components. Unlike a real chemical equation, such pathway diagrams indicate nothing about stoichiometry, concentrations, or changes in time (kinetics). That is, such diagrams are purely qualitative. Further, such pathways do not contain all possible binding interactions and rarely is justification given for excluding other known binding interactions. For example, in Figure 1, the presence of CHOP is presumed to be exclusively caused by activation of PERK, but it is well-known that other upstream pathways can also induce CHOP (the specific details are beyond our present scope) [67–70].

Often it is the case that the direct link between marker C and cell death is imprecise. For example, in Figure 1 it is empirically known that CHOP appears under some lethal cellular conditions [71], but how CHOP causes cell death is unknown. CHOP is a transcription factor that upregulates genes coding ER proteins, among others [72, 73]. Evidence suggests that CHOP activation in an injured cell overactivates and compromises ER function by overactivation of the translational machinery [74–76]. The steps between overactivating ER function or translation and the literal disintegration of a cell are unknown. In general, there is little, if any, attempt to delineate exactly how a hypothesized cell death marker C literally causes the cell to disintegrate. This holds for other cell death markers such as activated caspase 3, free radical species, and nitric oxide.

Additionally, the presence of a cell death marker is often treated as binary event: the presence of C equates with cell death and its absence with cell survival. Questions about rates and concentrations of marker C are rarely asked. For example, it is unknown how the concentration of CHOP correlates with the rate of cell death. Does more CHOP cause the cell to die faster?

In the brain ischemia field, there are relatively rare exceptions that seek to model intracellular events in terms of conventional rate and concentration expressions as found in classical biochemistry (e.g., [77, 78]). However, such approaches also possess important limitations. First, such analytical expressions are intended to apply to equilibrium systems and living cells are not at equilibrium. Close to- and far-from-equilibrium analytical treatments require more sophisticated mathematics [79]. Second, such expressions often require rate and binding constants that are either unknown, or not capable of being measured, and hence such models rely on making numerous assumptions whose veracity cannot readily be verified [75, 80] (see also discussion in supplementary text of [81]). Third, as with the qualitative schemes, such approaches cannot model all possible binding interactions and hence must necessarily model only a fraction of the relevant system. In spite of such limitations, classical equilibrium models are certainly a step up from qualitative pathways by providing quantitative insights at the equilibrium limit and potentially can be informative if the results are considered as modules or motifs embedded in a greater network.

Another point we discussed in detail elsewhere [45] and partially illustrated above is the fact that many different A → B → C pathways occur simultaneously in postischemic neurons, each of which correlates with cell death. Technically perhaps we should notate them as a set {A_1_ → B_1_ → C_1_, A_2_ → B_2_ → C_2_,…, A_*i*_ → B_*i*_ → C_*i*_}. With so many “cell death pathways” occurring simultaneously and all correlating with cell death, how can any one of them be singled out as “the cause” of cell death? It is this line of reasoning that exposes the inability of the inductive approach to assign causality. Additionally, there is always the possibility that some new pathway will be discovered tomorrow that might also correlate with cell death. The open-ended nature of the pathway approach exposes it as a form of induction.

Therefore, we conclude that these “pathway” diagrams create the illusion of explaining cell death without actually doing so. At best, they indicate qualitative events occurring inside an injured cell. In general, details of basic quantitative chemistry, such as concentrations, binding constants, and rates, are generally omitted from such schemes. Attempts to use classical chemistry formalism make the questionable assumption that far-from-equilibrium systems can be modeled by formalisms designed for equilibrium systems.

As mentioned above, inductive inferences usually have the form of generalizing from past to future events, or generalizing from observed to unobserved cases. By considering the case study of brain ischemia research, we find a third variant of inductive inference: attempting to model causality from cumulative specific observations. Such an approach results in a patchwork of observations cobbled together in an ad hoc fashion. Such schemes cannot intrinsically provide an understanding of cell death causality nor are they amenable to the type of quantitation that has allowed such success in the physical sciences. In short, such schemes represent a misapplication of inductive inference. Proof for this conclusion is to be found in the clinical trial failures that have resulted from this type of thinking.

## 8. A Deductive Approach to Cell Injury

In spite of the weakness of inductive theorizing, the associated empirical activities have uncovered phenomenology that provides a basis for developing alternative, deductive models of cell injury not limited by classical equilibrium and kinetic formulations. We devised such a model [7] by abstracting three phenomenological aspects of brain ischemia described above: (1) outcome is a function of both the (a) intensity (e.g., degree of blood flow reduction) and (b) duration of ischemia, (2) given specific intensity and duration parameters, cell outcome is binary: a given neuron either does or does not survive ischemia, and (3) the effect of injury inside the cell is multifactorial.

The multifactorial nature of cell injury is an alternate formulation of the same information used to construct the ischemic cascade. Instead of the futile attempt to link all the qualitative changes into a vast “meta” pathway diagram, one simply acknowledges that many events change in the injured cells. This then leaves open the issue of how these events causally link to outcome. Our thinking along these lines was influenced by a seminal notion put forth to account for how all ischemia-induced intracellular changes contribute to neuronal death. This is Wieloch's “sandwich model” [82] which posited two key notions. First, all of the separate damage mechanisms (the A_*i*_ → B_*i*_ → C_*i*_ described above), in some sense, add together to give the* total amount of damage*, *D*. Second, the cell contains an innate threshold, *D*_TH_, which is a specific amount *D*, below which the cell will recover and above which it will die.

The notion of summing all specific forms of damage to give the total damage is intuitive and natural. However, the concept of *D*_TH_ appears ad hoc unless it is recognized that injured cells not only experience damage but also induce stress responses, which are innate homeostatic mechanisms genetically coded in cells. Thus, a cell's ability to withstand damage will be a function of its innate stress responses. We expanded the sandwich model by positing that the individual stress responses also add together to give the* total induced stress response*, *S*. In this way, *S* replaces the idea of *D*_TH_.

Next, whether considered literally in terms of specific molecular damage products and stress responses, or thought of as the abstract totals, it will take time for *D* and *S* to accumulate, each eventually reaching some maximum value and then decreasing thereafter. Thus *D* and *S* are dynamical variables.

Finally, *D* and *S* do not magically appear; they are induced by an injury, whose magnitude can vary over some range. Intuitively, if injury magnitude is small, then we expect *D* and *S* to be small. If injury magnitude is large, we expect *D* and *S* to be larger. If injury magnitude is very large, we expect *D* to be very large but *S* to be small. This latter follows from the fact that stress responses are finite resources inside a cell, and therefore (1) will at some point saturate and (2) eventually succumb to damage [83].

We linked the above ideas into the following general process.

(1) Injury to a cell induces *D* and *S*.
(2) The magnitudes of *D* and *S* are driven by injury magnitude, *I*.
(3) As *I* increases, *D* increases, but *S* decreases.
(4) *D* and *S* are mutually antagonistic.

**Figure 2:**
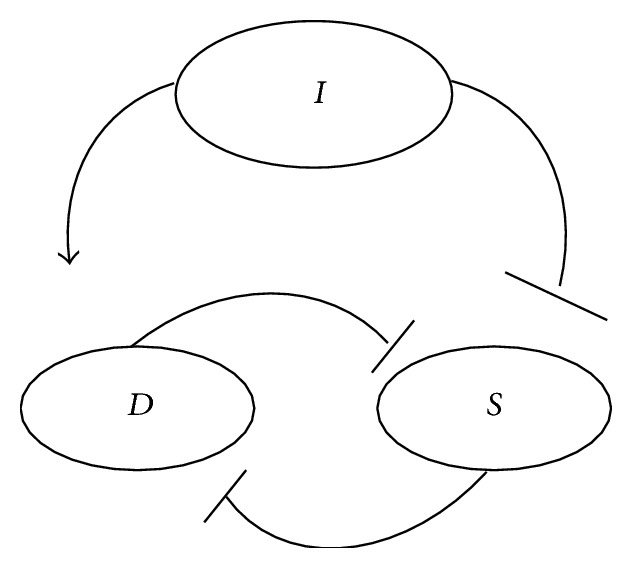
Circuit diagram underlying the deductive dynamical theory of cell injury. The core of the model is the mutual antagonism of *D* and *S*. *I* positively drives *D* and negatively drives *S*.

Figure 2 illustrates this process as a circuit diagram. The core of the circuit is the mutual antagonism of *D* and *S*. *I* is a positive driver on *D* and a negative driver on *S*. Qualitatively, how the model works is strikingly simple: if *S* > *D*,* the injured cell will recover, but if D* > *S*,* the injured cell will die*. By expressing these intuitive insights as a mathematical expression, a systematic and quantitative theory of cell injury was formulated.

## 9. A Deductive Model of Cell Injury

We elsewhere derived our mathematical model [7], which is summarized here. The model is expressed as a system of nonlinear ordinary differential equations (ODE). The simplest form of the model that captures the salient features of the circuit in Figure 2 is given by

(1) $$\begin{array}{c} \frac{dD}{dt}=\frac{{\left(c_{D}Ie^{I\lambda_{D}}\right)}^{n}}{{\left(c_{D}Ie^{I\lambda_{D}}\right)}^{n}+S^{n}}-D,\\ \frac{dS}{dt}=\frac{{\left(c_{S}Ie^{-I\lambda_{S}}\right)}^{n}}{{\left(c_{S}Ie^{-I\lambda_{S}}\right)}^{n}+D^{n}}-S, \end{array}$$

where

 
*n* is a Hill coefficient,
 
*c*_*D*_ is a measure of damage agent toxicity,
 
*λ*_*D*_ is how toxicity scales with *I*,
 
*c*_*S*_ is a measure of a cell's total stress response capacity,
 
*λ*_*D*_ is how total stress responses scale with *I*.

Equation (1) models the mutual antagonism of *D* and *S* by allowing the rate of formation of each to be inhibited by the other in the classical Hill equation form and assumes the intrinsic decays of *D* and *S* are uncoupled. The effect of injury magnitude, *I*, on *D* and *S*, respectively, is incorporated via the terms *c*_*D*_ *Ie* ^*λDI*^ and *c*_*s*_ *Ie* ^−*λsI*^. These expressions embody what we consider the simplest possible assumption of how *I* relates to *D* and *S*: *D* increases exponentially with *I*(*D* ∝ *e* ^*I*^), and *S* decreases exponentially with *I*(*S* ∝ *e* ^−*I*^).

Solving equation (1) under various parameter sets gives the time course of the *D* and *S* competition. The winner is determined by equation (1) in the form of the fixed points, or* attractor state*, (*D* ^*^, *S* ^*^). If *S* ^*^ > *D* ^*^, the cell recovers. If *D* ^*^ > *S* ^*^, the cell dies. The interpretation of equation (1) is to envision the uninjured cell at homeostatic steady state. Application of injury magnitude *I* is a “force” that displaces the cell from homeostasis, where the maximum displacement is given by the steady-state solution (*D* ^*^, *S* ^*^).

However, (*D* ^*^, *S* ^*^) is an intrinsically unstable state for the cell. A second expression is required to describe the resolution of the injury and the return of the cell back to a stable state. In the scope of our model, there are only two possible stable states to which the cell can return: (1) the preinjury homeostatic state, which is recovery, or (2) death, in which the cell no longer exists. In both cases, the final state approaches the initial state of an uninjured cell where (*D*, *S*) = (0, 0). The expression describing the decay from (*D* ^*^, *S* ^*^) to (0, 0) involves the simplest possible assumptions that: (1) this decay is exponential, and (2) the decay constant is the inverse of the magnitude (*D* ^*^ − *S* ^*^),

(2) $$\begin{array}{c} D\left(t\right)=D_{0}e^{-|D^{*}-S^{*}|t},\\ S\left(t\right)=S_{0}e^{-|D^{*}-S^{*}|t}, \end{array}$$

where *D*_0_ = *D* ^*^ and *S*_0_ = *S* ^*^.

Equation (2) is interpreted such that if *D* ^*^ > *S* ^*^, it gives the time it takes for the cell to completely disintegrate, and if *S* ^*^ > *D* ^*^ it gives the time it takes for the system to fully recover to the preinjury state.

Taken together, equations (1) and (2) describe the sequence of: (1) injuring the cell with injury magnitude *I*, followed by (2) the *D*/*S* competition, culminating in the solution (*D* ^*^, *S* ^*^), followed by (3) decay of the system either back to the pre-injury state (when *S* ^*^ > *D* ^*^) or to the death state (when *D* ^*^ > *S* ^*^). This sequence provides one possible dynamical interpretation of the circuit in Figure 2.

## 10. Theoretical Status of the Cell Injury Model

Here we very briefly comment on the status of this deductive model in the broader context of theoretical biology. We have rationalized the concepts of *D* and *S* as representing the global dynamics of an injury-induced intracellular network by invoking the principle that global network dynamics can constrain the many possible degrees of freedom of the nodes [84]. Intuition and empirical evidence let us recognize that outcome after acute injury is binary: survival or death. Thus, the complex network of intramolecular changes induced by acute injury must be constrained by only these two options. Given the wide variety of acute injury conditions leading to cell survival or death, these are not unreasonable assumptions and at present must be treated as hypotheses requiring empirical validation.

We discussed above the limitations of applying classical rate and equilibrium expressions to model cell injury, and we comment how our model fits into this broader framework. The ideas behind *D* and *S* are novel. We consider them to represent coarse-grained emergent phenomena, analogous in some sense to the coarse-grained or ensemble-averaged variables in classical thermodynamics. However, unlike classical thermodynamics, the systems the model is intended to describe, injured cells, are nonequilibrium. Our model implies, but does not explicitly account for, the underlying physical processes that maintain the nonequilibrium state. Thus, at present, the model itself must be considered phenomenological in the sense we have no explicit link to any specific theory of nonequilibrium processes. However, the formal analogy to, if not implicit dependence on, gene regulatory network dynamics [85] leaves open possibilities for deducing a more firm theoretical foundation in the future.

## 11. Theoretical Implications of a Deductive Theory of Cell Injury

In the current biomedical research arena, each injury or disease state is taken as a separate entity. Researchers who study each injury are experts in the phenomenology of that system within the limits of experimental animal models and clinical phenomenology. This gives rise to distinct biomedical fields. For example, the study of brain ischemia is a different field than the study of cardiac ischemia, each having its own professional societies, journals, NIH Institutes, conferences, medical specialties, and so forth.

Clearly the requirements of technical and expert knowledge give rise to this specialization. But it is an unbalanced situation because there has not been a corresponding unity binding the diverse biomedical fields. The deductive view implied by equations (1) and (2) offers a counter balance to the effects of specialization and provides an example of unifying a variety of forms of biological injury in a consistent framework. Equations (1) and (2) allow us to think of cell injury in generic terms, abstracted from biological context. Cell injury becomes the general process depicted in Figure 2, whereby injury magnitude, *I*, drives two variables, total damage, *D*, and total induced stress responses, *S*, in opposite directions. This mitigates focus on specific cell biology with respect to the cause of injury-induced cell death.

This gives rise to the most important theoretical implication of equation (1): the cause of cell death is abstracted away from biological specifics and replaced with a simple unifying concept:* cell death is caused when D* ^*^ > *S* ^*^. The specific molecular details of *D* and *S* will certainly vary from system to system and impact technical aspects of the science, but the underlying theoretical unity remains. Roughly speaking, this is similar to the concept of temperature. Temperature is independent of the specific molecular details of a system: 25°C is the same temperature whether measured in water, oil, air, or a solid object. Similarly, for any given specific injured system, if *D* ^*^ > *S* ^*^, no matter what molecules instantiate this relationship, according to equation (1), the system can deterministically die. Thus, the cause of cell death in any injured system is now simple, comprehendible, and potentially applicable across a wide variety of specific types of injury applied to specific cell types.

## 12. Empirical Implications of a Deductive Model of Cell Injury

To empirically test equation (1) means altering how cell injury is measured and analyzed. There must be a move away from conflating specific biological changes with cell death causation. Instead,* injury-induced biological changes should be considered as markers* of *D and S*. To measure *D* and *S* requires an inordinate amount of specific biological information, which is possible to acquire with -omics technology [86]. However, the qualitative biological details are secondary to the quantitative magnitudes of change used to estimate *D* and *S*. We recently discussed measuring *D* and *S* [86] and will not repeat those ideas here.

To understand the required shift in empirical thinking means understanding the link between equation (1) and experimental designs. Equation (1) predicts specific time courses for *D* and *S*. Therefore, real *D* and *S* time courses need to be measured and compared to those predicted by equation (1). If they match, equation (1) constitutes a valid theory of cell injury; otherwise equation (1) needs to be modified to match the experimental data. Such an iterative cycle of comparing empirical data to equation predictions and altering equations as necessary is precisely the scientific process of using a deductive model and is routine, for example, in physics. Once it is established how to measure *D* and *S* time courses, and a suitable expression is determined, whether equation (1) or some other ODE system, then the goal is to use the *D* and *S* time courses to empirically measure the parameters in equation (1).

The equation parameters become the means to represent specific injury systems, where an* injury system* is the specific cell type plus the specific form of damage. We discuss elsewhere [7, 86] that the values of *c*_*D*_ and *λ*_*D*_ represent the relative lethality of some specific* damage agent*, which is the specific form of injury with intensity *I*. The values of *c*_*S*_ and *λ*_*S*_ quantify the strength of a specific cell type's intrinsic stress response potential. It is through determining the parameters of a specific injury system that the nonlinear cell injury theory may then be applied to the goal of therapeutics, as discussed in the next section.

When the numerical values of the injury system parameters are empirically measured, the system is then termed “fully determined.” The term “fully determined” is a technical term that means that the empirically determined system parameters can be plugged into the equation of the system and solved mathematically, thereby revealing the full system dynamics. At this point,* the scientific problem has been solved*, and the knowledge can be applied, for example, towards the invention of therapeutic applications.

Thus, the empirical measurement of cell injury is deeply altered by use of a deductive mathematical framework and will more resemble the performance of science in physics, materials science, or other mathematical-based sciences, where numerical parameter values are routinely used to characterize specific systems, such as heat capacity of liquids, tensile strength of metals, or refractive index of solids.

## 13. Implications of a Deductive Model for Systematic Therapeutics

With respect to therapeutics, a central idea emerges from the deductive theory of cell injury:* The science of solving a specific cell injury system is distinctly different from applying that knowledge once acquired*.

This is a key point to emphasize because one of the major assumptions of the current inductive mode of thinking is that discovering a correlation between “pathway *x*” and cell death is synonymous with therapy. Conflating a specific molecular pathway with cell death causality implies that therapy* is* the manipulation of that pathway. Thus, it has become the hallmark of legitimacy for biomedical studies to inhibit “pathway *x*,” manipulate pathway “*x*” to prevent cell death, and thereby “prove” the cell death mechanism.

This is no mere theoretical consideration, but is the criterion that determines which manuscripts are accepted for publication, which grants are funded. While this logic has produced thousands of instances of success in animal experimental models, it has wholly failed in human clinical trials, and biomedical communities struggle to account for this glaring disparity. We have elsewhere extensively criticized this approach [84] and even offered a plausible explanation, using our deductive theory, of how this situation might have arisen [7]. Here we emphasize that, from a deductive point of view, solving the system, that is, fully determining the parameters of the theoretical equations, is a distinctly different activity from how the solutions to the equations may be applied.

Ironically, fully determining equation (1) for a specific injury system offers a benefit that is completely unsuspected within the mainstream inductive mindset:* every possible state of the system becomes known, and therefore, every potential state of therapy also becomes known*. To explain this, we first digress briefly on how to solve an ODE.

## 14. Digression on Solving an ODE

Although the technical details of solving an ODE are beyond the scope of this paper, we briefly summarize the nature of the answers one gets when solving an ODE system. Equation (1) encodes an infinity of *D* and *S* time courses, all of which obey the rule which equation (1) constitutes. Figure 2 is a visual depiction of the rule which equation (1) constitutes. When equation (1) is solved for a specific injury system this extracts a* subset* of time courses from equation (1), those which describe all possible states of the specific system defined by the numerical values of the parameters. Different parameters specify different subsets of time courses, analogous to how different addresses specify different buildings. Once a given set of parameters is specified, the resulting subset of time courses extracted from the ODE system comes in the form of a hierarchy, as now explained.

**Figure 3:**
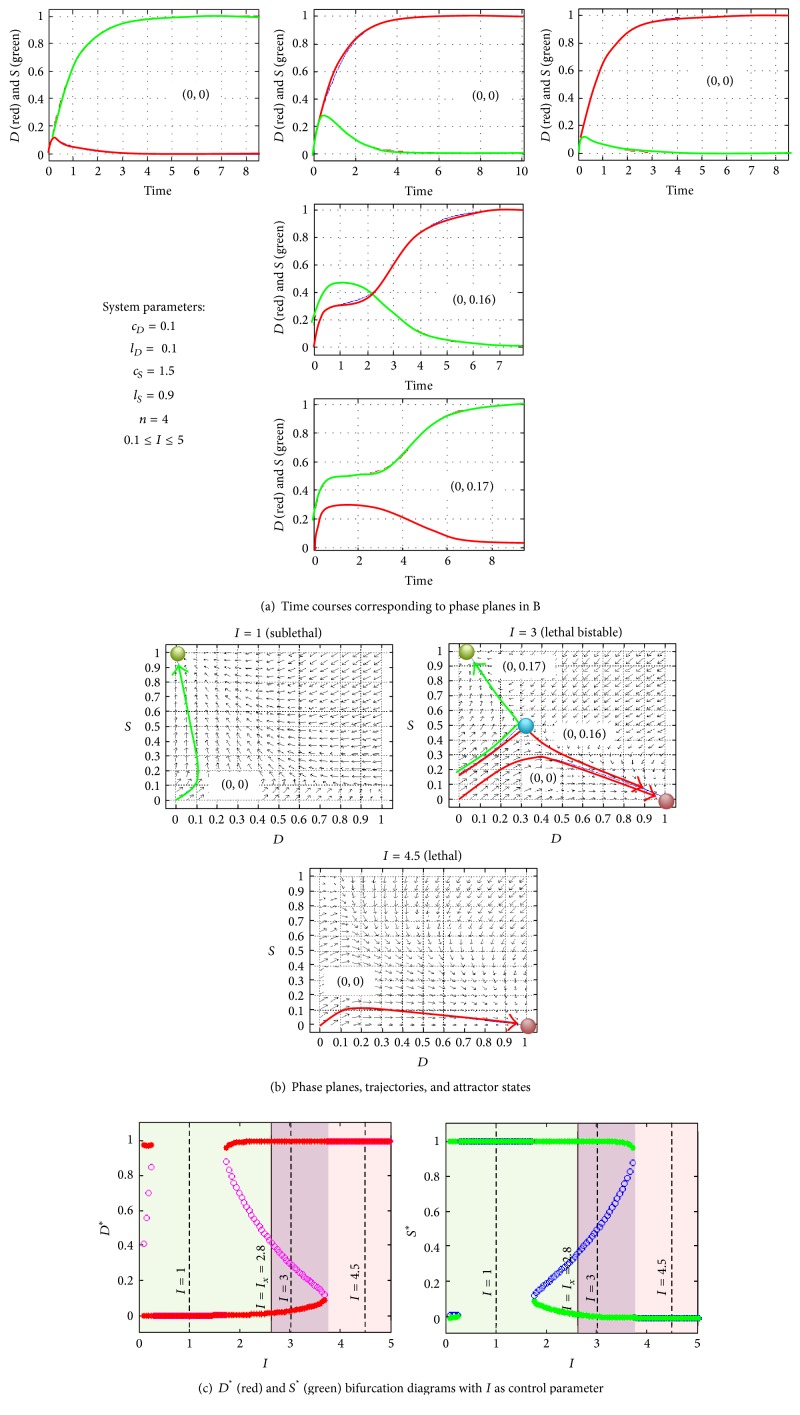
ODE solutions form a hierarchy. (a) The bottom of the hierarchy consists of a single pair of *D* and *S* time courses. These are the empirically accessible objects of the theory and would be tested against real *D* and *S* time courses to determine how well the predicted time courses fit experimentally measured time courses. The three columns of time courses derive from the phase planes in B and represent monostable sublethal, bistable lethal, and monostable lethal time courses, respectively. The monostable time courses and top bistable time course are from initial conditions (*D*_0_, *S*_0_) = (0, 0). The middle bistable time course is from initial conditions (0, 0.16) and indicates that preactivating stress responses to 16% of their maximum value are not sufficient to flip state. However, at 17% of stress responses preactivation, the system flips state and survives an insult that would be lethal from initial conditions (0, 0). (b) The middle of the hierarchy consists of phase planes showing trajectories at all possible initial conditions for a given set of parameters. Trajectories are converted to pairs of *D* and *S*time courses by well-established methods (e.g., Runge-Kutta). The phase planes shown correspond to the *D* and *S* time course pairs in A. The middle phase plane is bistable. The survival and death attractors are shown as green and red circles, respectively. The three trajectories on the middle phase plane correspond to the above time courses, as indicated. (c) The top level of the hierarchy is a bifurcation diagram. When the control parameter for the bifurcation diagram is injury magnitude, *I*, we call the resulting bifurcation diagram an* injury course*. The phase planes in B are indicated by dashed lines, labeled accordingly. The system parameters in A give rise to a doubly bistable injury course. The bifurcation diagrams shown would constitute the “answer” to and would fully characterize the injury system represented by the parameter set in A. In our deductive theory, the cause of cell death is always *D* ^*^ > *S* ^*^. The injury course (bifurcation diagram) becomes the way to formulate any injury system, and it provides a basis for a comprehensive and systematic approach to therapeutics. The bistable region is indicated by the open circles, which are unstable repeller fixed points. The areas marked by purple boxes are the* therapeutic region*, those injury states where it is possible in principle to flip state and prevent a system that would normally die from dying. That equation (1) not only predicts bistability but also provides a systematic and quantitative understanding of it is perhaps the most important novel contribution of the nonlinear dynamical theory of cell injury.

The bottom of the hierarchy is a single pair of *D* and *S* time courses which are obtained by specifying all six equation parameters, plus the initial conditions of the time courses (Figure 3(a)). A single pair of *D* and *S* time courses corresponds to a real injury system that can be empirically measured.

The middle level is called a phase plane (Figure 3(b)). A phase plane is specified by the exact same six parameter values, but the initial conditions can take on any value in the plane. A phase plane therefore encodes many different pairs of *D* and *S* time courses (Figure 3(b)). The key feature of a phase plane, which links together all the associated time courses, is the phase plane's* attractor state*. For equation (1), attractors are notated (*D* ^*^, *S* ^*^). The attractor is a point on the phase plane where the time courses end (i.e., the steady state reached by each time course). If there is only one attractor, the phase plane is called “monostable,” and all the time courses end on that attractor. If there are two attractors, the phase plane is “bistable”: some time courses end on one, some on the other attractor. More than two attractors correspond to a multistable system. We have shown previously that equation (1) exclusively outputs monostable or bistable phase planes [7] and so do not concern ourselves with multistability here.

The top level of the hierarchy is a bifurcation diagram, which is obtained by holding five of the six parameters constant and varying only one parameter. The parameter which is varied is called the* control parameter*. In equation (1), injury magnitude *I* is the control parameter (Figure 3(c)). A bifurcation diagram is a plot of the value of the attractor states versus the value of *I*. In the scope of the dynamical cell injury model, the bifurcation diagram corresponds to measuring the injury system at different magnitudes of *I*, which we call an* injury course* [7, 86]. So, as a phase plane is a “bundle” of time courses linked by common attractor states, a bifurcation diagram links a bundle of phase planes via the control parameters. A bifurcation diagram is a bundle of bundles of time courses.

To summarize, equation (1) can output (1) a specific pair of *D* and *S* time courses (representing a measurable system), or (2) many related time courses forming a phase plane, and (3) many related phase planes forming a bifurcation diagram. Fully determining a specific injury system allows one to calculate any of these entities. These mathematical entities define all possible states of the system, including those amenable to therapeutics.

## 15. Deductive Therapeutics

We previously discussed therapeutics in the context of equation (1) [7, 86] and summarize the main result here. Therapeutics implies two possibilities: (1) slowing the death of an injured system that is fated to die or, (preferably) (2) preventing the death of an injured system that is fated to die. Equation (1) accommodates both possibilities, predicts precisely when either possibility will occur, and provides a quantitative measure of how to prevent the cell from dying. We focus here on preventing the death of an injured system that is fated to die.

The possibility of preventing cell death occurs when equation (1) outputs bistable solutions, again, meaning there are two attractors for a given set of parameters. One attractor represents recovery, while the other attractor represents death. If the injury magnitude, *I*, is lethal (meaning *D* ^*^ > *S* ^*^ from initial conditions *D*_0_ = 0 and *S*_0_ = 0), but the system is bistable, there exists a possibility to divert the system from the prodeath attractor to the prosurvival attractor and thereby prevent the cell from dying (Figure 3(b)). We call this process “flipping state” from a death outcome to a survival outcome. The ability to flip state is revealed by the bifurcation diagram, which shows the range of injury magnitudes that produce bistable attractor states. The bistable range of injury magnitudes is indicated in Figure 3(c) by the portions of the plots with open circles, which are unstable repellers that accompany bistable attractors. The lethal bistable regions are indicated by the ranges inside the purple boxes: here the system would die from (*D*_0_, *S*_0_) = (0, 0), but since the system is bistable, there is the possibility of flipping state to a survival outcome. To the right of this region, there is no possibility to flip state because the system is monostable lethal.

The implications of bifurcation diagrams such as Figure 3(a) are profound. They reveal all possible outcomes in the injury system for all possibly magnitudes of injury. In addition, the bifurcation diagram encodes the quantitative dynamics of total damage, *D*, and the total stress responses, *S*, that link directly to the specific biology. This is a highly systematic and quantitative understanding wholly absent from inductive models of cell injury. The bifurcation diagram is akin to a phase diagram that shows when the system undergoes major qualitative transitions. For equation (1), there are only four possible qualitative states of the injured system's dynamics: (1) monostable, nonlethal, (2) monostable, lethal, (3) bistable, nonlethal, and (4) bistable, lethal. There are no other possibilities for outcome. It is the bistable, lethal states where it is potentially possible to flip state and cause a system that would otherwise die to survive.

No inductive model offers such a complete and systematic insight into cell injury and therapy, let alone one that can be generalized across diverse biological injury conditions.

## 16. Personalized Medicine

Our final topic briefly considers a possible application of the deductive approach to therapy in the context of the emerging practice of “personalized medicine.” Personalized medicine is the idea that physicians will tailor-make therapies on the fly for individual patients [87], because it is now possible or will be possible in the near future to obtain full genomic [88] and proteomic [89] information on individual patients. At present, genomic information is used to screen for risk of genetic diseases [90] and also to guide in drug efficacy [91]. However, it is only at early research stages for applications in diagnosis or treating of acute injury, such as stroke [92]. Our deductive approach suggests the possibility of real-time application in the clinic for acute injuries such as stroke or myocardial infarction.

Already, the -omics are being pursued as a means to obtain more sophisticated and informative biomarkers of the course and progression of acute injuries such as stroke [93, 94] and myocardial infarction [95]. But can this information be used for therapeutic purposes? We suggest that it may be possible to couple real-time, patient-acquired -omics information with equation (1) to determine (1) where on the *D* and *S* time courses a specific patient's disease progression falls, (2) whether or not the patient is at a bistable injury magnitude, and (3) if so provide an indicator of the intensity of therapy needed to “flip state” in the patient's injured tissue.

One can imagine a technology that will take -omics data as input via serum or cerebrospinal samples, perhaps coupled with other information such as fMRI in stroke, or ultrasound in myocardial infarction, use this input data to solve in real time equations (1) and (2) (or whatever their empirically-validated equivalent turns out to be), and output bifurcation diagrams, phase planes, and time courses specific for the patient.

Such technology could revolutionize the treatment of acute injuries such as stroke and myocardial infarction by showing the course and prognosis of individual acute injuries to guide physician treatment decisions. These are tangible benefits that are simply not possible to envision using the current inductive pathway mode of thinking.

## 17. Concluding Comments

We contrasted inductive and deductive approaches to cell injury, which is starkly illustrated by comparing Figures 1 and 2. Each approach necessitates its own theoretical, empirical, and analytical methods. Inductive approaches have driven biomedical studies to the present. Developments in the physics of complex systems, coupled with the rise of the -omics era in biology, provide a basis to develop a deductive approach such as described above. There are two concerns to close this paper.

First, because of the differences between the inductive and deductive approaches, there is the possibility of a Kuhnian “communication breakdown,” where scientists practicing each approach “speak through each other” and do not communicate [16]. This situation must be avoided. Inductive work to the present, in spite of its limitations, has laid the foundation to develop any future deductive approaches. There is thus an intimate complimentary that has the possibility of evolving into a culture similar to that in modern physics and engineering where theoretical and empirical physicists cooperate and work side by side to solve scientific problems, from which engineers can use the solutions to design novel technologies.

On the other hand, current biomedicine is one-sided because inductive approaches dominate biomedicine. The weakness of inductive biomedical science is evident in the large record of clinical trial failures for such important injuries as stroke, cardiac arrest, myocardial infarction, and so on. The inductive game of “guess the pathway” has become uncritically accepted as the mainstream in many biomedical fields. This way of thinking has grown out of proportion relative to its utility and must be reined in. Inductive approaches to acute injury are reaching a point of diminishing returns: the largest phenomenological effects have been discovered, and only smaller and smaller effects remain to be discovered. If treating the largest effects has not been successful, then confidence is diminished that treating ever smaller quantitative effects will be successful.

Balance can be restored by the application of deductive and formal models of cell injury. Our model offers one such approach. We cannot claim, lacking evidence at present, that equation (1) is* the* correct description of cell injury dynamics. However, it certainly serves as a starting point for formulating deductive cell injury models. The logic it exposes, particularly the link between bistability and therapeutics, provides serious motivation for pursuing this approach. By moving in this direction it will bring to biomedicine the approaches that have led to such great scientific and technological success in the physical sciences.
